# A marine-derived small molecule induces immunogenic cell death against triple-negative breast cancer through ER stress-CHOP pathway

**DOI:** 10.7150/ijbs.70975

**Published:** 2022-04-11

**Authors:** Haiyan Wen, Yinxian Zhong, Yuping Yin, Kongming Qin, Liang Yang, Dan Li, Wulin Yu, Chenjie Yang, Zixin Deng, Kui Hong

**Affiliations:** Key Laboratory of Combinatorial Biosynthesis and Drug Discovery, Ministry of Education, School of Pharmaceutical Sciences, Wuhan University, Wuhan 430071, PR China.

**Keywords:** triple-negative breast cancer, sesterterpene, endoplasmic reticulum stress, C/EBP-homologous protein, oxidative stress, immunogenic cell death

## Abstract

Although triple-negative breast cancer (TNBC) is the most refractory subtype among all breast cancers, it has been shown to have higher immune infiltration than other subtypes. We identified the marine-derived small molecule MHO7, which acts as a potent immunogenic cell death (ICD) inducer through the endoplasmic reticulum (ER) stress-C/EBP-homologous protein (CHOP) pathway, to treat TNBC. MHO7 exerted cytostatic and cytotoxic effects on TNBC cells at an IC_50_ of 0.96-1.75 µM and suppressed tumor growth with an approximately 80% inhibition rate at a dose of 60 mg/kg. In 4T1 cell tumor-bearing mice, 30 mg/kg MHO7 inhibited pulmonary metastasis with an efficacy of 70.26%. Transcriptome analyses revealed that MHO7 changed the transcription of genes related to ribosome and protein processes in the ER. MHO7 also triggered reactive oxygen species (ROS) generation and attenuated glutathione (GSH) levels, which caused excessive oxidative stress and ER stress via the PERK/eIF2α/AFT4/CHOP pathway and led to cell apoptosis. ER stress and ROS production facilitated the release of ICD-related danger-associated molecular patterns (DAMPs) from TNBC cells, which activated the immune response *in vivo*, as indicated by the release of antitumor cytokines such as IL-6, IL-1β, IFN-γ, and TNF-α, increases in CD86^+^ and MHC-II dendritic cells and CD4^+^ and CD8^+^ T cells and a decrease in regulatory T cells (Tregs). These results reveal that MHO7 triggers an aggressive stress response to amplify tumor immunogenicity and induce a robust immune response. This synergistic effect inhibits primary breast cancer growth and spontaneous metastasis in TNBC, providing a new strategy for TNBC treatment.

## Introduction

Breast cancer has replaced lung cancer as the most common cancer worldwide, with approximately 2.3 million new cases and 685,000 deaths in 2020 1. Among several subtypes of breast cancer, triple-negative breast cancer (TNBC), which is characterized by a lack of estrogen, progesterone, and HER2/neu expression, is the most refractory subtype due to its extremely high aggressiveness and metastasis 2. The lack of specific carcinogenic biomarkers makes patients with TNBC insensitive to hormones or targeted molecular therapies 3. Chemotherapy remains the main treatment for patients with metastatic TNBC, resulting in a median overall survival of 12-18 months and less than 30% five-year survival 4. It has been reported that TNBC has higher tumor-infiltrating lymphocyte levels than other subtypes, resulting in improved clinical outcomes and sensitivity to chemotherapy 5. However, for patients with advanced TNBC, existing clinical agents are far from satisfactory. Thus, there is an urgent clinical need to develop new therapeutic agents for TNBC.

Taking advantage of tumor immunogenicity, immunotherapy and novel chemotherapy have achieved recent success clinically 6. During this process, tumor antigens play a significant role in promoting the immune response and recruiting sufficient T cells to exert a cytotoxic effect. In many cases, immunosuppression inevitably progresses with tumor development, and one reason is that regulatory T cells (Tregs) suppress the immune response and pave the way for the immune escape of tumor cells 7. An essential consideration in enhancing the effect of immunotherapy is to increase the release of neoantigens in tumors and amplify the innate immune response 8. Tumor immunogenic cell death (ICD) can produce neoantigens when tumor cells are killed by an external stimulus. This strategy can transform a nonimmunogenic environment to an immunogenic environment and mediate the antitumor immune response 9.

ICD is a special kind of apoptosis characterized by the ability of apoptotic cells to induce an adaptive immune response, which provides a promising strategy for the development of therapeutic agents with oncological applications 10. ICD is characterized by the release of damage-associated molecular patterns (DAMPs), which include calreticulin (CRT), adenosine-5′-triphosphate (ATP), high mobility group protein B1 (HMGB1), and heat shock proteins (HSPs) 11. ICD has been verified to be dependent on endoplasmic reticulum (ER) stress and reactive oxygen species (ROS) 12. Although some compounds can cause ER stress and oxidative stress, they are unable to induce DAMP release. It was reported that the ER stress inducer (thapsigargin or tunicamycin) or cisplatin alone was incapable to elicit the translocation of CRT from the lumen of the ER to the cell surface to induce CRT exposure and ICD 13. These observations confirmed that ER stress and oxidative stress are endogenous stimuli for ICD and that DAMPs are also required for ICD to activate anticancer immunity. In fact, screening work has been performed on approximately nine hundred compounds, including clinically used medicines, to identify novel ICD inducers for cancer treatment, of which only septacidin was selected, apart from some known ICD inducers 14. Thus, it is necessary to develop more novel agents to induce effective ICD for cancer treatment.

The ER is an important organelle that acts as a general inducer of ICD under stress conditions 15. ER stress-activated pathways, which are also called the unfolded protein response (UPR), and particularly the protein kinase RNA-like endoplasmic reticulum kinase (PERK)-mediated pathway, are essential for the production of many ICD-related DAMPs. It was reported that CRT exposure required PERK activation and the secretory pathway during hypericin-based photodynamic therapy (Hyp-PDT) treatment 16. When sustained ER stress exists, the UPR fails to restore homeostasis and leads to PERK-dependent induction of the C/EBP-homologous protein (CHOP) pathway, which plays a critical role in ER stress-mediated apoptosis 17.

During apoptosis, immunogenic DAMPs are released from apoptotic cells to the tumor microenvironment (TME). These DAMPs play different roles in initiating the tumor-specific immune response. In the early stage of apoptosis, CRT and HSPs translocate from the ER to the cell surface, providing an 'eat me' signal to activate phagocytosis by dendritic cells (DCs) 16. Then, ATP is secreted from apoptotic cancer cells and acts as a 'find me' signal to promote DC maturation and the secretion of the stimulatory cytokine interleukin (IL)-1β 18. During the last stage, HMGB1 is released from the nucleus to stimulate intense inflammation, and the proinflammatory cytokine IL-6 is released, facilitating antigen presentation from DCs to T cells 19. Then, tumor-specific cytotoxic T lymphocytes are activated, accompanied by the release of immune-promoting cytokines such as IFN-γ and TNF-α to kill tumor cells 20.

Marine microorganisms, especially marine fungi, are promising resources for the discovery of novel drug compounds 21. Ophiobolins (Ophs), which are widely found in marine fungi or sponges, are sesterterpene compounds with a unique tricyclic 5-8-5 ring system 22. Our previous study demonstrated that marine-derived ophiobolin O exerted an antitumor effect on breast cancer cells through the AKT or MAPK pathway 23, 24. In recent years, we have paid attention to another compound known as MHO7 (6-epi-ophiobolin G), which shows vigorous antitumor activity 25. MHO7 showed a robust effect on non-TNBC cell lines, such as MCF-7, T47D and ZR-75-1 cells, with IC_50_ values of 1.24~7.14 μM, and it was identified as a potent estrogen receptor (ERα) degrader in breast cancer 26. However, MHO7 also inhibits TNBC with no ERα to target, and its potential mechanism remains unclear.

Here, we demonstrated that MHO7 exerted a potent antitumor effect on TNBC *in vitro* and* in vivo*. MHO7 induced severe ER stress through the PERK/eIF2α/ATF4/CHOP pathway and caused cell cycle arrest at the G0/G1 phase in TNBC cells. Additionally, MHO7 induced ROS generation, which contributed to ER stress-mediated apoptosis. Such severe ER stress and ROS production induced by MHO7 facilitated the release of ICD-related DAMPs, which further induced ICD *in vivo* and mediated a robust antitumor immune response, as evidenced by the release of antitumor cytokines, the activation of mature DCs and high levels of CD8+ T cells but deceased Tregs, ultimately leading to the inhibition of tumor growth and metastasis. Our findings first revealed the potential of MHO7 in tumor treatment through the induction of ICD, providing a promising candidate for clinical therapeutic strategies against TNBC.

## Material and Methods

### Reagents and antibodies

MHO7 was extracted and purified (>96%) from the fermentation products of *Aspergillus ustus* and its structural elucidation and biosynthesis had been described previously 27. Doxorubicin hydrochloride (catalog #HY-15142) was obtained from MedChem Express (Shanghai, China). β-Tublin (catalog #AF7011), p-PERK (catalog #DF7576), p-eIF2α (catalog #AF3087), ATF4 (catalog #DF6008), CHOP (catalog #DF6025), HSP70 (catalog #AF5466), Bax (catalog # AF0120) and Cleaved-Caspase 3 (catalog #AF7022) antibodies were obtained from Affinity Biosciences (Changzhou, China). β-actin (catalog #20536-1-AP), BCL2 (catalog #12789-1-AP), caspase 3 (catalog #19677-1-AP), HMGB1 (catalog #66525-1-Ig), CRT (catalog #10292-1-AP), ATP1A1 (catalog #14418-1-AP) antibodies were purchased from Proteintech Group (Rosemont, IL, USA). Ki67 (catalog #GB111141) and BiP (catalog #A4908) antibodies were acquired from Servicebio (Wuhan, China) and ABclonal Technology Co., Ltd (Wuhan, China), respectively. CD8α (catalog #383302) and FOXP3 (catalog #ab215106) antibodies were purchased from ZEN-BIOSCIENCE (Chengdu, China) and Abcam (Cambridge, MA, USA), respectively. Anti-rabbit IgG (H+L) (catalog # SA00001-2), anti-mouse IgG (H+L) (catalog # SA00001-1) were acquired from Proteintech Group (Rosemont, IL, USA). 7-AAD staining solution (catalog #559925), CD16/CD32 Pure 2.4G2 (catalog #553141), APCCy7-CD45 (catalog #557659), mouse T Lymphocyte Subset Antibody Cocktail (catalog #558391), PE-CD86 (catalog #561963), PECy7-CD11c (catalog #561022), APC-MHC-II (catalog #562367), and APC-FOXP3 (catalog #560401) for flow cytometry were all purchased from BD Biosciences (New York, MA, USA). eBioscience™ FOXP3/Transcription Factor Staining Buffer Set (catalog #00-5523-00) was obtained from Thermo Fisher Scientific Inc. (Waltham, MA, USA). N-acetylcysteine (NAC) was obtained from Shanghai yuanye Bio-Technology Co., Ltd (Shanghai, China). Annexin V-FITC Apoptosis Detection Kit and PI/RNase Staining Buffer were purchased from BD Biosciences (New York, MA, USA). Fluo-4 AM, ROS detection kit (DCFH-DA), GSH and GSSH detection kit, ATP assay kit, and membrane and cytosol protein extraction kit were all purchased from Beyotime Institute of Biotechnology (Jiangsu, China). CCK-8 assay kit was acquired from Dojindo (Japan). PAGE Gel Fast Preparation Kit was obtained from Epizyme Biotech (Shanghai, China) and JC-1 was purchased from MedChemExpress (Shanghai, China).

### Cell culture

Human breast cancer cell lines MDA-MB-231, MDA-MB-468 and the epithelial cells of normal breast MCF-10A were cultured in Dulbecco's modified Eagle's medium (Gibco, ThermoFisher, Waltham, MA, USA), and murine breast cancer cell line, 4T1 was cultured in RPMI-1640 medium (Gibco, ThermoFisher, Waltham, MA, USA), with 5% CO_2_ at 37 °C. All culture media contained 10% fetal bovine serum (AusGeneX, Australia), 100 μg/mL of streptomycin, and 100 U/mL penicillin (Gibco, ThermoFisher, Waltham, MA, USA). All cells were purchased from CCTCC (China Center for Type Culture Collection, Wuhan, China).

### Cell viability assay

Cell viability was assayed by CCK-8 solution. Briefly, cells were seeded in 96-well plates until attachment and incubated with a series concentration of MHO7 for 12, 24, or 48 h. After that, CCK-8 solution was added to each well and incubated for about 30-60 min. Then, cell viability was measured at 450 nm using a spectrophotometer (TECAN, Infinite 200 PRO Switzerland).

### Apoptosis assay

For apoptosis assay, cells were cultured in 6-well plates and treated with MHO7 for 24 h. After that, cells were collected and then resuspended in a 1X binding buffer. Before incubating 15 min at RT in the dark, 5 µl of FITC Annexin V and 5 µl PI were added in cells. Finally, a flow cytometer (CytoFLEX, Beckman Coulter) was used to measure the apoptosis of cells.

### Real-time quantitative PCR

Total RNA was isolated from MHO7-treated cells or tumors by RNA sample Total RNA Kit from TIANGEN BIOTECH Co., Ltd., (Beijing, China), and RNA integrity was assessed using the RNA Nano 6000 Assay Kit of the Bioanalyzer 2100 system (Agilent Technologies, CA, USA). After that, 1μg RNA was subjected to reverse transcription using HiScript III-RT SuperMix for qPCR (+gDNA wiper) (Vazyme, Jiangsu, China). Primers for real-time PCR were designed with the Primer-BLAST web tool (Table S1). Analysis of real-time PCR was performed by Taq Pro Universal SYBR qPCR Master Mix (Vazyme, Jiangsu, China). Relative mRNA gene expression was calculated using a 2^-ΔCt^ method.

### RNA-sequencing and data analyses

Total RNA was isolated from tumors or cells as above. RNA-sequencing (RNA-seq) was next accomplished by Beijing Novogene Bioinformatics Technology Co., Ltd. (Beijing, China). Sequencing libraries were generated using NEBNext® UltraTM RNA Library Prep Kit for Illumina® (NEB, USA) following the manufacturer's recommendations, and index codes were added to attribute sequences to each sample. Differential expression analysis of the samples was performed using the DESeq2 R package (1.20.0). Corrected P-value of 0.05 and absolute foldchange of 2 were set as the threshold for significantly differential expression. Enrichment analysis of differentially expressed genes was implemented by the clusterProfiler R package. Heatmaps were drawn by TBtool (v.1.09) 28.

### Western blot analysis

Cells were rinsed with PBS and lysed in RIPA buffer containing a protease inhibitor cocktail for about 30 min. After that, the lysate was collected and centrifuged for 15 min at 12000 rpm and the protein in the supernatant was detected by BCA protein assay kit (Servicebio, Wuhan, China). The cell lysates mixed with protein loading buffer and loaded in SDS-polyacrylamide gels then transferred to PVDF membranes (Millipore, Billerica, MA). Then, membranes were blocked and incubated with specific primary antibodies. Before visualizing by ECL system (biosharp, Hefei, China), membranes were incubated with horseradish peroxidase (HRP)-conjugated secondary antibodies.

### Cell cycle detection

Cell cycle was measured by PI/RNase Staining Buffer according to manufacturer's guideline. Briefly, cells were cultured in 6-well plates and exposed under MHO7 for 24 h. After washing twice with PBS, cells were fixed with ethanol at 4 °C overnight. Then, collected the cells and stained with PI/RNase Staining Buffer for 30 min at 37 °C and detected by flow cytometry.

### ROS generation

For the detection of intracellular ROS generation, cells were seeded in 6-well plates until attachment, followed by the pretreatment with or without 4 mM NAC for 1 h and incubation with various concentrations of MHO7 for 24 h. After that, cells were washed with PBS and incubated with a DCFH-DA probe for 30 min at 37 °C. Then, PBS was used to wash away excess probes and quantify the level of ROS by flow cytometry.

### GSH/GSSG assay

Intracellular GSH and GSSG level was measured by GSH/GSSG assay kit according to the manufacturer's guide. After treatment with 4 mM NAC for 1 h or MHO7 for 24 h, cells were collected to detect the intracellular GSH and GSSG by spectrophotometer.

### Detection of mitochondrial membrane potentials (MMPs)

The changes of MMPs were detected by JC-1. Briefly, cells were seeded in 6-well plates and treated with MHO7 for 24 h. After that, 10 μL of JC-1 was added to cells and incubated for 30 min at 37 °C in the dark. Then, cells were collected and washed twice with PBS and resuspended in PBS. Finally, cells were analyzed by flow cytometry.

### Cytosolic Ca^2+^ levels detection

Intracellular-free Ca^2+^ was measured using Fluo-4AM. Briefly, cells were cultured in 6-well plates and incubated with different concentrations of MHO7 for 24 h. Then, cells were collected and incubated with Fluo-4AM for 30 min at 37 °C and immediately detected by flow cytometry.

### Extraction of the cell membrane and cytoplasmic protein

The cell membrane and cytoplasmic protein were isolated using the Surface and Cytoplasmic Protein Reagent Kit according to manufacturers' instructions. First, cells were washed with PBS and lysed with reagent A and centrifuged to extract the protein in cytoplasmic. Next, add reagent B to extract membrane protein. The extracted protein was analyzed by western blot.

### CRT exposure and HMGB1 release

CRT and HMGB1 expression were detected by immunofluorescence. TNBC cells were seeded into 35 mm dishes and cultured until attachment. After the treatment of MHO7 or DOX (2 μM) for 24 h, cells were washed twice in PBS and fixed with 4% paraformaldehyde for 15 min and blocked by 3% Albumin Bovine (Biofroxx, 4240, Germany) for 30 min. Then, CRT or HMGB1 polyclonal antibody was diluted (1:100) and incubated at 4 °C overnight. After that, cells were labeled with Alexa Fluor 488 or cy3 conjugated secondary for 1 h at room temperature. Subsequently, cells were stained with DAPI for 5 min and observed.

### ATP detection

The release of ATP was detected by the ATP assay kit. After the treatment with MHO7 for 24 h, the supernatants of MDA-MB-231 and 4T1 cells were collected at 0, 2, 4, 8, 12, or 24 h and analyzed according to the manufacturer's instructions. The luminescence was detected by Infinite200Pro (TECAN).

### Transfection

MDA-MB-231 cells in a 6-well plate were transfected with double-stranded siRNA against CHOP (Genecreate, Wuhan, China) for 24 h using Lipofectamine 2000 reagents (Invitrogen, Grand Island NY). The sequences of CHOP siRNA are 5′-AGAGCCCUCACUCUCCAGAUUTT-3′ and 5′-AAUCUGGAGAGUGAGGGCUCUTT-3′. According to the manufacturer's instruction, 50 nM siRNA was used. After 48 h, cells were treated with 3 μM MHO7 for 24 h. Then, cells were collected for further analyses.

### Animal experiments

All animal experiments were approved by the Institutional Animal Care and Use Committee (IACUC) of the Institute of Model Animals of Wuhan University. Female BALB/c nude mice or BALB/c mice (about 4-6 weeks old) were all obtained from and maintained in the Experimental Animal Center of Wuhan University. Xenografts Models were established by female BALB/c nude mice, with MDA-MB-231 cells (5 ×10^6^/mice) subcutaneously inoculated at the axilla of back legs. Balb/c-nude mice were divided into five groups (n=6) randomly: (1) Vehicle *i.p.*; (2) M-30 group (30 mg/kg MHO7, *i.p.*, once every two days); (3) M-60 group (60 mg/kg MHO7, *i.p.*, once every two days); (4) DOX group (5 mg/kg DOX, *i.p.* once every three days); (5) combination group (2.5 mg/kg DOX + 10 mg/kg MHO7). The doses of MHO7 are determined by pre-experiment, and dose of DOX is according to Sun et al. 29.

Then, 4T1- cells (about 2 ×10^6^) were inoculated into the mammary fatty pad of BALB/c mice to establish a primary breast cancer model. The tumor volume was calculated twice a week according to the formula: volume=1/2 (length×width^2^). When the tumors reached 50-100 mm^3^, the mice were randomly divided into several groups (n=10). According to the results of pre-experiment in BALB/c nude mice, we adjusted the dose of MHO7 and DOX and separated the mice into five groups: (1) Vehicle* i.p.*; (2) M-10 group (10 mg/kg MHO7, *i.p.*, once every two days); (3) M-30 group (30 mg/kg MHO7,* i.p.*, once every two days); (4) DOX group (2.5 mg/kg DOX, *i.p.,* once every three days). This dose is according to Yeh et al. 30; (5) combination group (2.5 mg/kg DOX + 10 mg/kg MHO7). The tumor size and weight of mice were monitored every other day until giving final treatment. After that, mice were sacrificed then several tissues, including important organs and tumors, were separated for further analysis. At the first, the middle, and the last period during administration, whole blood and serum samples from BALB/ c mice were collected for hematology and biochemistry analysis, respectively.

For vaccination assay, cells were treated with vehicle or MHO7 (3 μM) for 48 h, respectively, to obtain dying 4T1 cells. Then, the dying cells (1×10^6^) above were injected subcutaneously into the left flank of BALB/c mice (6-week-old female; n=10). After 7 days, untreated 4T1 cells (2×10^6^) were injected into the right flank of the vaccinated mice. Tumor growth and survival were monitored for the following weeks. Animals were euthanized when the maximum diameter of tumor exceeds 15 mm or the human endpoints was reached.

### ELISA assay for the Cytokine detection

The determination of treatment-induced cytokine release was used in ELISA assay. The serum was collected after the treatment, and the concentration of pro-inflammatory cytokines such as IFN-γ, TNF-α, IL-1β, and IL-6were measured by ELISA kit (Neobioscience Technology Co, Ltd, Beijing, China).

### Histological analysis

The tumor and major organs (lung, liver and kidney) were harvested from mice and fixed for hematoxylin and eosin (H&E) or immunohistochemistry (IHC) staining. H&E staining was used to evaluate the possible toxicity to important organs and cytotoxicity to tumors. Images were captured using an Olympus microscope. Image-Pro Plus version 6.0 software (Media Cybernetics, Inc., Rockville, MD, USA) was used to assess the area and density of the dyed region, and the integrated optical density (IOD) value of the IHC section. The mean densitometry of the digital image was designated as representative staining intensity of Ki67 and cleaved caspase3. The signal density of the tissue areas from five randomly selected fields were counted in a blinded manner and subjected to statistical analysis 31. Immunofluorescence staining was performed to detect the mean fluorescence intensity of, CRT, HMGB1, CD8 and FOXP3 were analyzed by Image J (version 1.8.0).

### Immune response detection

To investigate the immune effect *in vivo*, flow cytometric analysis was performed to detect the single cells extract from the spleens of BALB/c mice. After the treatment for 2 weeks, the mice were sacrificed, and spleens were collected and digested to prepare single cells suspensions. Before flow cytometry, T lymphocytes were stained with anti-CD3 -PECY7, anti-CD4-PE, anti-CD8-FITC antibodies; DCs maturation was detected by staining with anti-CD11c-PECY7-PC7, anti-CD86-PE, and anti-MHC-II-APC and anti-CD86-APC antibodies; Tregs were stained by anti-CD3 -PECY7, anti-CD4-PE, and anti- FOXP3- APC after fixed and permeabilized.

### Statistics

Data were shown as Mean ± SD (or ±SEM where indicated). Statistically significant differences were calculated using Tukey-Kramer test. Statistical significance is displayed as: ns, not significant; *P<0.05; **P< 0.01; ***P< 0.001. All data were analyzed using GraphPad Prism (GraphPad Software Inc, San Diego, California).

## Results

### Antitumor effect of MHO7 on TNBC *in vitro* and *in vivo*

**Figure 1:**
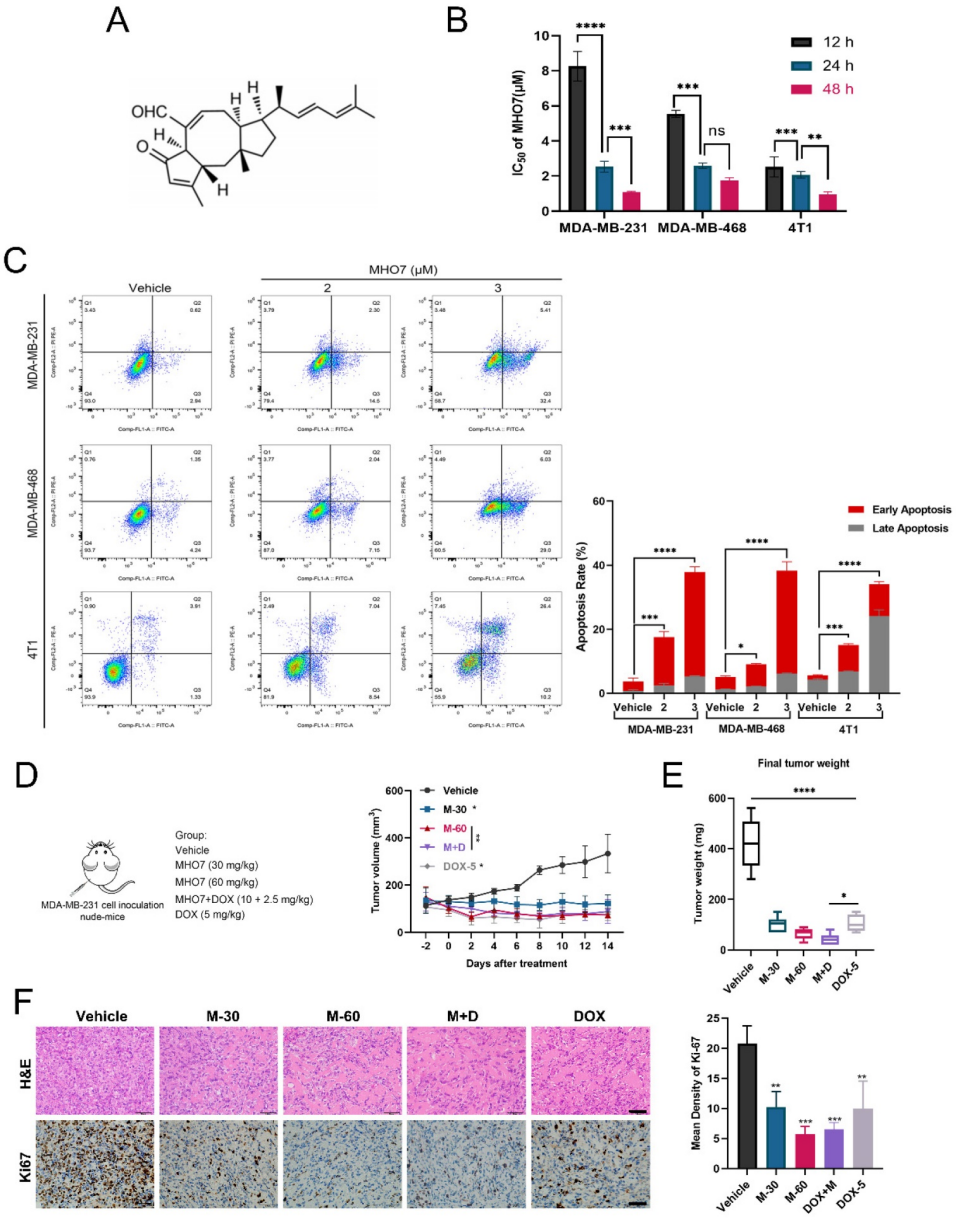
** Antitumor effect of MHO7 on TNBC *in vitro* and *in vivo*. (A)** The chemical structure of MHO7. **(B)** The IC_50_ value of MHO7 on TNBC cell lines at 12, 24, and 48 h. **(C)** The percentages of apoptosis cells were measured by flow cytometry under MHO7 treatment at 24 h. **(D)** Tumor growth was measured in mice. Data was presented as mean ± SEM. (n=6). **(E)** Final tumor weight was measured in each group. Data was presented as mean ± SEM. (n=6). **(F)** Tumor tissue was analyzed by H&E staining and immunohistochemistry analysis of Ki67. Scale bars 50 µm. (n=3). *P <0.05; **P < 0.01; ***P <0.001; and**** *P* < 0.0001.

To explore the inhibitory effect of MHO7 on tumor cell proliferation, MDA-MB-231, MDA-MB-468, 4T1 and epithelial MCF-10A cells were treated with various concentrations of MHO7 for 12, 24, or 48 h. Fig. 1A shows the chemical structure of MHO7. As shown in Fig. 1B, MHO7 significantly decreased the proliferation of TNBC cell lines in a time-dependent manner, and the half-maximal inhibitory concentration (IC_50_) values of MDA-MB-231, MDA-MB-468, and 4T1 cells at 48 h were 1.07 ± 0.12, 1.75 ± 0.16 and 0.96 ± 0.14 μM, respectively. However, MHO7 showed lower cytotoxicity against MCF-10A cells than TNBC cells, with an IC_50_ of 3.03 ± 0.19 μM at 48 h (Fig. S1A-S1B). Flow cytometry showed that MHO7 induced significant apoptosis in TNBC cell lines in a dose-dependent manner (Fig. 1C). Additionally, cleaved caspase 3 was overexpressed, whereas the ratio of the antiapoptotic protein Bcl-2 to the proapoptotic protein Bax was suppressed compared that in vehicle cells, suggesting that MHO7 induced significant apoptosis in TNBC cells (Fig. S1C-S1D). Given the encouraging results *in vitro*, we subsequently established MDA-MB-231 xenograft tumor-bearing models to evaluate the antitumor effect of MHO7 *in vivo*. Tumor-bearing mice were divided into 5 groups and treated with 30 and 60 mg/kg MHO7 (M-30 and M-60), 5 mg/kg DOX (DOX-5) 29 or the combination of 10 mg/kg MHO7 and 2.5 mg/kg DOX (M+D). Mice treated with MHO7 alone were intraperitoneally (*i.p.*) administered 30 mg/kg or 60 mg/kg for 2 weeks, and the mean tumor volume was reduced by 63% and 79%, respectively. Treatment with 5 mg/kg DOX every third day *i.p*. caused a 73% reduction in tumor growth but led to death in 4/6 of the mice. Tumor inhibition in the combination group reached 77% (Fig. 1D). Accordingly, mice in the M-60 and M+D groups exhibited the lowest final tumor weights at sacrifice (Fig. 1E). During the treatment period, 30 mg/kg MHO7 was well tolerated, with no significant body changes or damage to liver and kidney tissue, but body weight reductions occurred in the 60 mg/kg MHO7 and DOX groups (Fig. S1E-S1F). The inhibition of tumor growth was paralleled by severe damage to tumor tissue, as shown by H&E staining, and a reduction in tumor proliferation, as measured by Ki67 expression (Fig. 1F). Overall, these data indicated that MHO7 showed effective antitumor activity in TNBC and showed good safety* in vitro* and* in vivo*, which provided a strong basis for further investigation.

### MHO7 induced ER stress and cell cycle arrest in TNBC cells

**Figure 2:**
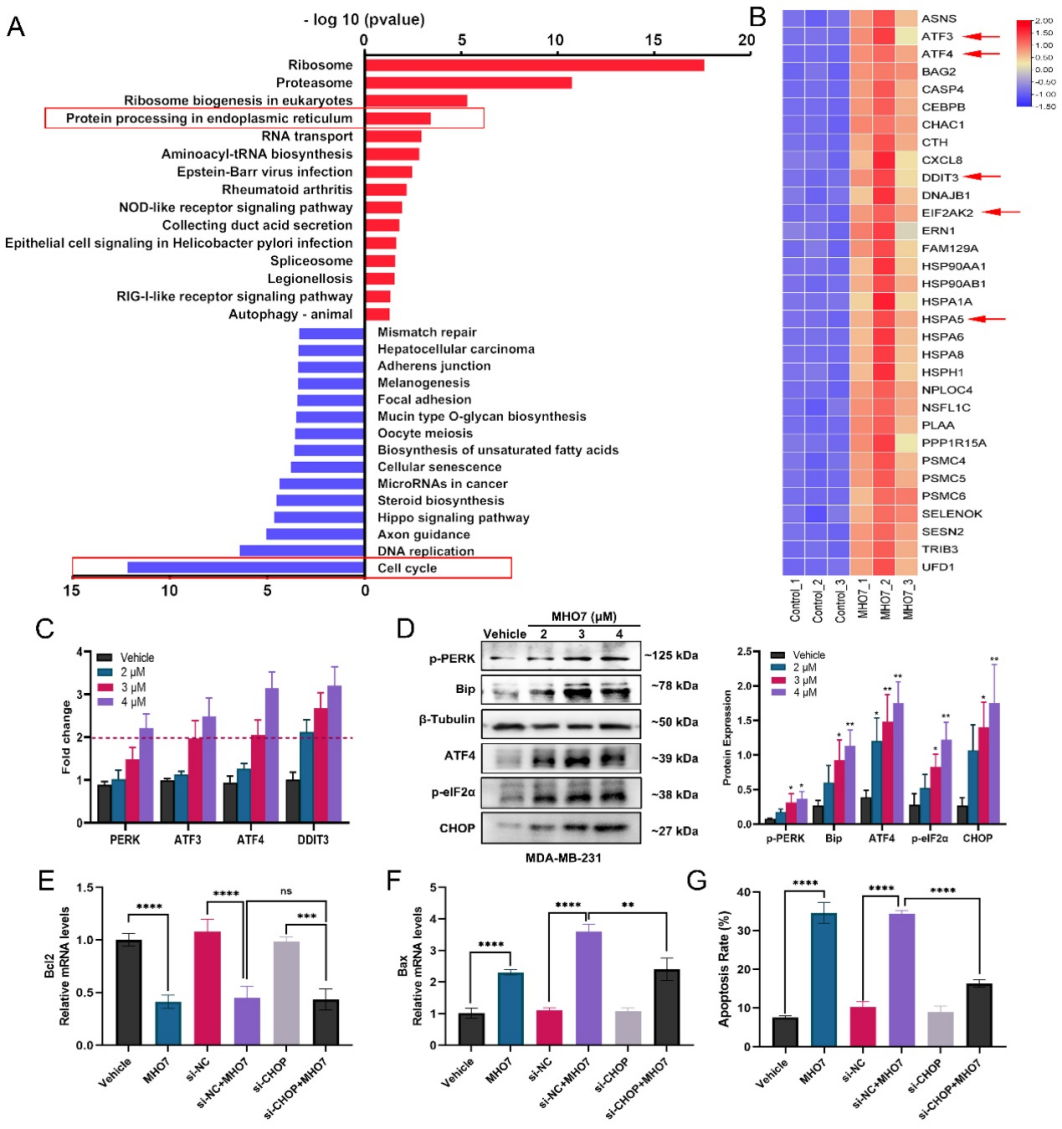
** MHO7 induced ER stress and cell cycle arrest in TNBC cells. (A)** The top KEGG pathway enrichment was analyzed by RNA-seq. The red highlighted upregulated pathways, and the blue highlighted downregulated pathways. **(B)** The heat map of DEGs related to ER stress. **(C)** ER stress-regulated genes in MDA-MB-231 cells were detected by qRT-PCR under MHO7 treatment. **(D)** The expression of BiP/p-PERK/p-eIF2α/ATF4/CHOP were measured by western blot under MHO7 treatment in MDA-MB-231 cells. **(E)** Bcl-2 was detected by qRT-PCR when si-CHOP or MHO7 (3 µM) treatment. **(F)** Bax was detected by qRT-PCR when si-CHOP or MHO7 (3 µM) treatment. **(G)** Representative apoptosis rate was measured by flow cytometry under si-CHOP or MHO7 (3 µM) treatment. *P <0.05; **P < 0.01; ***P <0.001; and ****P < 0.0001; ns=no significant.

To further explore the potential mechanisms underlying MHO7 treatment in TNBC, RNA-seq was performed on MHO7 (3 μM)-treated MDA-MB-231 cells. Compared with those in the untreated group, there were 1059 differentially expressed genes (DEGs), including 471 upregulated genes and 588 downregulated genes (|log2FC|>1, *p*<0.05) (Fig. S2A). Notably, pathway enrichment analyses of DEGs showed particular enrichment of upregulated genes associated with protein processing in ribosomes or the proteasome and related to protein processing in the ER, while downregulated genes were enriched in the regulation of the cell cycle or DNA replication (Fig. 2A). Genes in the pathway “protein processing in endoplasmic”, such as ATF3, ATF4, DDIT3, EIF2AK2, and HSPA5, were uniquely upregulated, and genes in the pathway “cell cycle” or “DNA replication”, such as CDK1, CDK2, and E2F1, were significantly downregulated in MHO7-treated cells compared to control cells (Fig. 2B and Fig. S2B). Flow cytometry showed that the cell cycle was significantly blocked at the G0/G1 phase by MHO7 (Fig. S2C). These results suggested that ER stress and cell cycle arrest played major roles in MHO7-induced apoptosis in TNBC.

To confirm these results related to ER stress, qRT-PCR was performed to measure UPR genes that contribute to ER stress. The mRNA expression levels of PERK, ATF3, ATF4, and DDIT3 were more than 1.5-fold upregulated in MDA-MB-231 cells treated with >3 μM MHO7, which indicated that MHO7 could induce ER stress via the PERK/ATF4/CHOP pathway (Fig. 2C). The western blot results showed that MHO7 significantly activated the protein expression of BiP, p-PERK, p-eIF2α, ATF4 and CHOP in TNBC cells (Fig. 2D and Fig. S2D). As one of the important regulators of the ER stress-mediated apoptosis pathway, CHOP functions as a transcription factor to control apoptosis under ER stress conditions 32. To further confirm the role of CHOP in MHO7-induced apoptosis, we used siRNA to knock down CHOP expression. We found that CHOP knockdown could also suppress the expression of Bax after treatment with MHO7 but did not affect Bcl-2 expression, suggesting that CHOP mediated MHO7-induced apoptosis partly by regulating Bax (Fig. 2E-2F). Another apoptotic protein, cleaved-caspase 3, was upregulated when cells were exposed to MHO7, and transfection of si-CHOP reduced the level of MHO7-induced cleaved-caspase 3 (Fig. S3A-S3B). Moreover, si-CHOP obviously decreased the cell apoptosis rate induced by MHO7 (Fig. 2G and Fig. S3C). These results demonstrated that MHO7 caused severe ER stress and induced ER stress-mediated apoptosis through the PERK/eIF2α/ATF4/CHOP pathway and that CHOP played an essential role in MHO7-induced apoptosis.

### MHO7-induced ROS generation contributed to ER stress-mediated apoptosis

**Figure 3:**
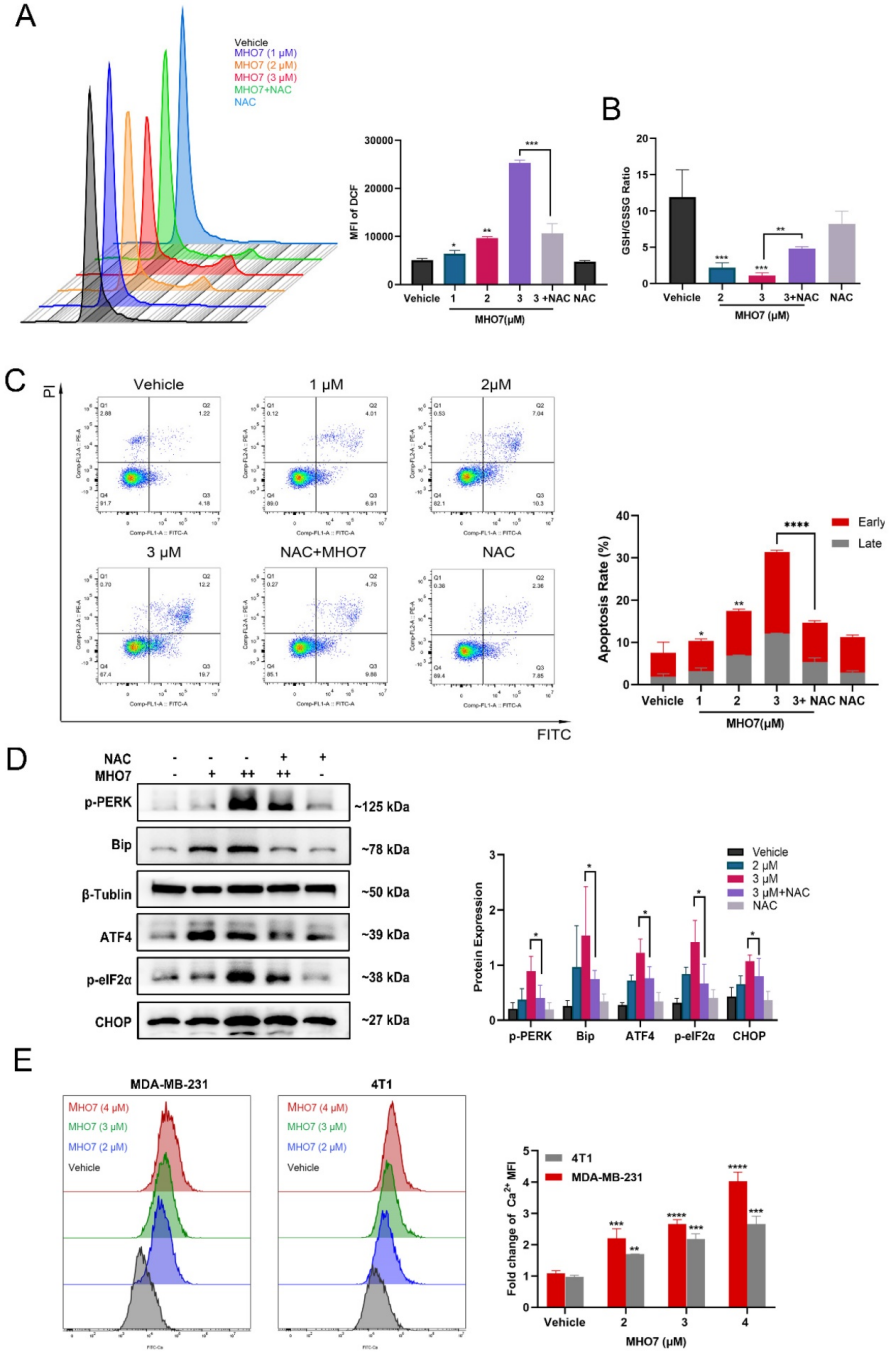
** MHO7-induced ROS generation contributed to ER stress-mediated apoptosis. (A)** The ROS level was detected by flow cytometry when treated with MHO7 for 24 h or pretreated with NAC (4 mM) for 1 h in MDA-MB-231 cells. **(B)** GSH /GSSG ratio of MDA-MB-231 cells was detected under the treatment of MHO7 (2, 3 µM) and pretreatment of NAC (4 mM). **(C)** The percentages of apoptosis cells were measured by flow cytometry when treated with MHO7 for 24 h or pretreated with NAC (4 mM) for 1 h in MDA-MB-231 cells. **(D)** ER stress-related proteins were measured by western blot under the treatment of MHO7 (+: 2 µM; ++:3 µM) or pretreated with NAC (4 mM) in MDA-MB-231 cells. **(E)** Intracytoplasmic calcium ionic was detected by flow cytometry after the treatment of MHO7 in MDA-MB-231 and 4T1 cells. *P <0.05; **P < 0.01; ***P <0.001; and ****P < 0.0001.

It has been reported that the induction of ER stress is always accompanied by oxidative stress, and it is a highly interrelated process that regulates multiple signaling pathways in cells 33. To study the effect of MHO7 on the induction of oxidative stress, we used N-acetylcysteine (NAC) as a ROS scavenger and examined ROS generation and GSH levels. As shown in Fig. 3A and Fig. S4A, treatment with MHO7 caused higher ROS levels than the vehicle or NAC treatment in MDA-MB-231 and 4T1 cells, respectively. Moreover, MHO7 decreased the GSH/GSSG ratio (Fig. 3B), suggesting that MHO7 disrupted intracellular redox homeostasis. To gain further insight into the relationship between ROS generation and ER stress-mediated apoptosis, we examined apoptosis and ER stress-associated protein expression after cotreatment with NAC and MHO7. Notably, flow cytometry showed that NAC alleviated MHO7-induced apoptosis (Fig. 3C). In addition, pretreatment with NAC significantly decreased the expression of the ER stress-regulated proteins p-PERK, BiP, p-eIF2α, ATF4 and CHOP in MDA-MB-231 cells and 4T1 cells, indicating that ROS contributed to ER stress-induced apoptosis (Fig. 3D and Fig. S4B). The ER lumen is considered to be the major intracellular Ca^2+^ storage compartment, playing a critical role in Ca^2+^ homeostasis 34. Thus, cytoplasmic Ca^2+^ was measured by flow cytometry. MHO7 increased cytoplasmic Ca^2+^ levels (Fig. 3E). The depletion of the ER Ca^2+^ is followed by the rapid accumulation of Ca^2+^ inside the mitochondrial matrix through the uniporter system, and both cytoplasmic Ca^2+^ and ROS generation lead to mitochondrial damage 35. MMPs were reduced by MHO7 treatment (Fig. S4C). These results confirmed that MHO7 enhanced ROS generation, which contributed to ER stress-mediated apoptosis, leading to the disruption of Ca^2+^ homeostasis and mitochondrial dysfunction.

### MHO7 induced the release of DAMPs from TNBC cells

**Figure 4:**
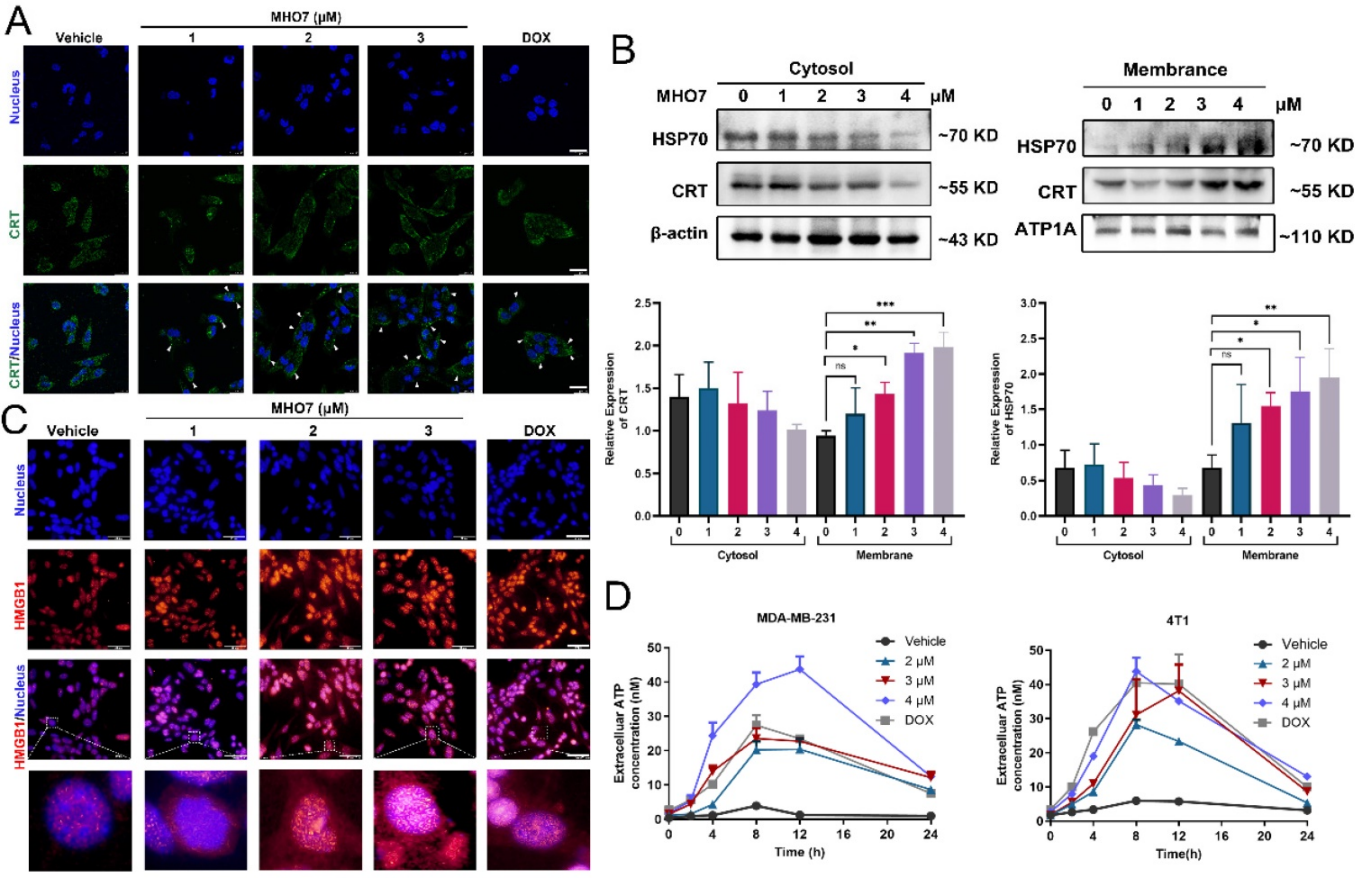
** MHO7 induced the release of DAMPs from TNBC cells. (A)** CRT translocation was analyzed by immunofluorescence staining after exposure to MHO7 or DOX (2 µM). Scale bars 25 µm. **(B)** Expression of CRT and HSP70 proteins in the cell cytosol and the cell membrane were measured by western blot under treatment with MHO7. **(C)** HMGB1 exposure was detected by immunofluorescence staining after MHO7 or DOX (2 µM) treatment. Scale bars 50 µm. **(D)** ATP was detected in the cell supernatant of MDA -MB-231 and 4T1 cells after MHO7 or DOX (2 µM) treatment for 24 h. *P <0.05; **P < 0.01; ns: no significance.

A notable feature of ICD inducers is their ability to induce ER stress and oxidative stress, accompanied by enhanced DAMP levels and an activated immune response 36. DAMPs are released from dying cells. Since DOX is an ICD inducer and is widely used for chemotherapy of breast cancer, we used it as a positive control for MHO7 37. As shown in Fig. 4A and 4C, the immunofluorescence results showed that CRT was translocated to the cell membrane from the cytoplasm, and HMGB1 translocated from the nucleus to the cytoplasm in response to MHO7 in a dose-dependent manner, and the effect of MHO7 was stronger than that of DOX at a high dose. Further analysis by western blotting also showed that MHO7 treatment increased CRT and HSP70 levels in the cell membrane but decreased them in the cytosol (Fig. 4B). An increase in ATP in the culture supernatant was detected at a concentration of approximately 2 to 18-43 nM within 12 h of treatment with various concentrations of MHO7 or DOX, followed by a gradual decrease at 24 h (Fig. 4D). Collectively, these results indicated that MHO7 could trigger the release of DAMPs to induce ICD.

### MHO7 suppressed tumor growth and pulmonary metastasis in tumor-bearing mice

**Figure 5:**
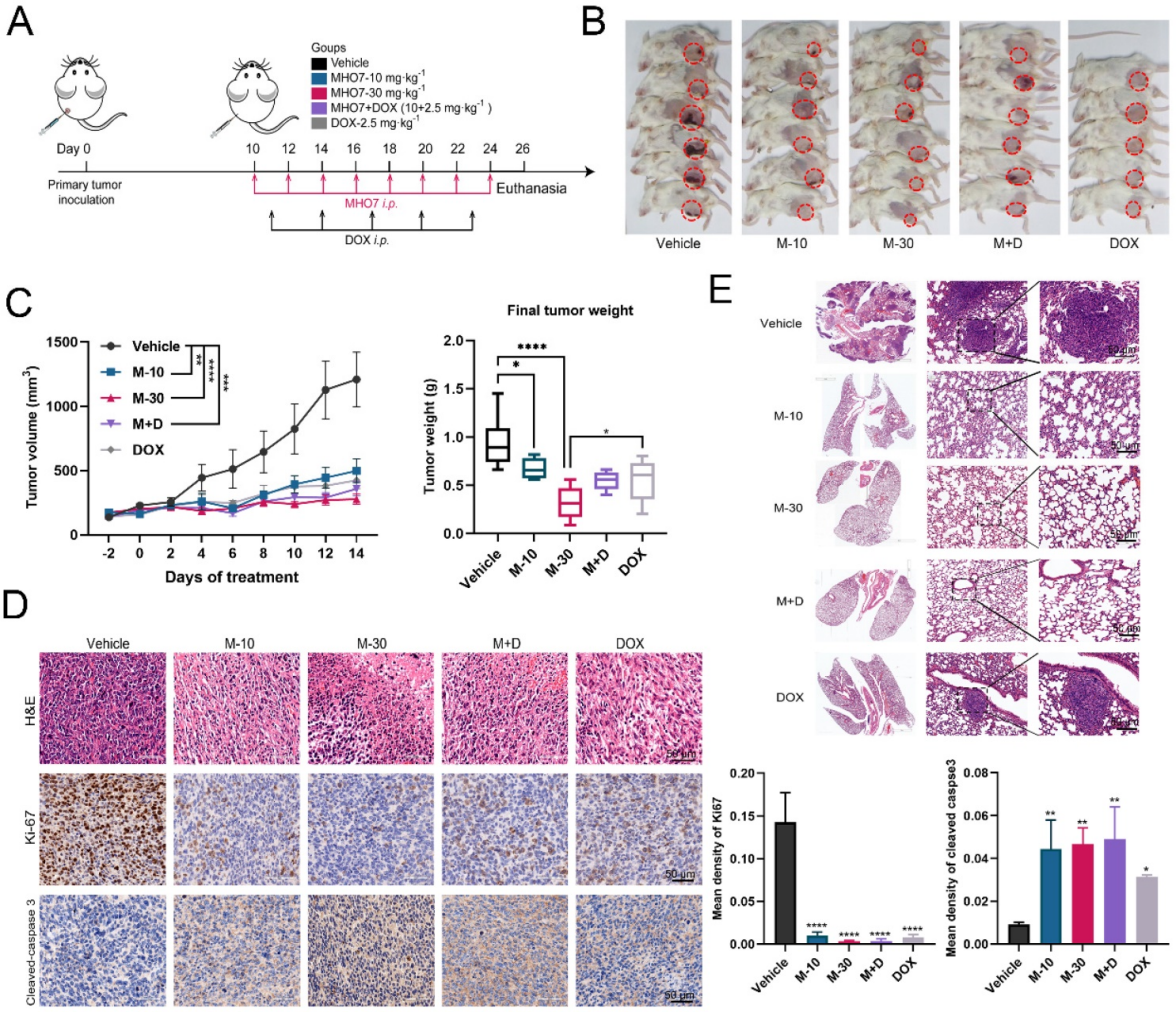
** MHO7 suppressed tumor growth and pulmonary metastases in tumor-bearing mice. (A)** Diagram of procedure for mouse tumor model. **(B)** The Observation of the primary tumors. **(C)** Tumor growth and the final tumor weights were measured in mice. (n=6). Data was presented as mean ± SEM. **(D)** Ki67 and cleaved caspase 3 expression in tumor tissues were analyzed by H&E staining and IHC staining. Scale bar, 50 µm. **(E)** Lung tissues in mice was analyzed by H&E staining, and the tumor areas were indicated by the black circle. *P <0.05; **P < 0.01; ***P <0.001; and ****P < 0.0001.

To confirm the ICD-inducing function and antitumor effect of MHO7 *in vivo*, BALB/c mice with an intact immune system were implanted with 4T1 cells in mammary fat. Because 5 mg/kg DOX caused 4/6 death and 60 mg/kg MHO7 reduced the weights of nude mice, we adjusted the dose of MHO7 and DOX and separated the mice into five groups: (1) Vehicle *i.p.;* (2) M-10 group (10 mg/kg MHO7, *i.p*., once every two days); (3) M-30 group (30 mg/kg MHO7, *i.p.*, once every two days); (4) DOX group (2.5 mg/kg DOX, once every three days, according to Yeh et al. 30); and (5) combination group (2.5 mg/kg DOX + 10 mg/kg MHO7) (Fig. 5A). Fig. 5B shows the primary breast tumors in mice, and 1 death occurred in the DOX group. Compared to that in the vehicle group, the tumor size in the MHO7- or DOX-treated groups began to shrink 4 days after administration. Surprisingly, mice treated with M-10, DOX and M+D showed similar inhibition rates of 56.27%, 62.81%, and 61.43%, respectively, and the M-30 group exhibited better outcomes (70.26%) than the other groups (Fig. 5C). MHO7 markedly inhibited the expression of Ki67 and increased cleaved caspase 3 levels, suggesting that MHO7 causes severe damage and low proliferation or high apoptosis in TNBC cells (Fig. 5D). At the end of the treatment, lung tissues were collected, and H&E staining was performed to verify the appearance of lung metastases. As shown in Fig. 5E, mice in the vehicle group and DOX group exhibited lung metastases, but no visible lung metastases were observed in the MHO7-treated groups (M-10, M-30, and M+D groups). These data demonstrated that MHO7 markedly suppressed primary breast tumor growth and pulmonary metastasis in tumor-bearing mice.

### MHO7 activated the antitumor immune response in mice

**Figure 6:**
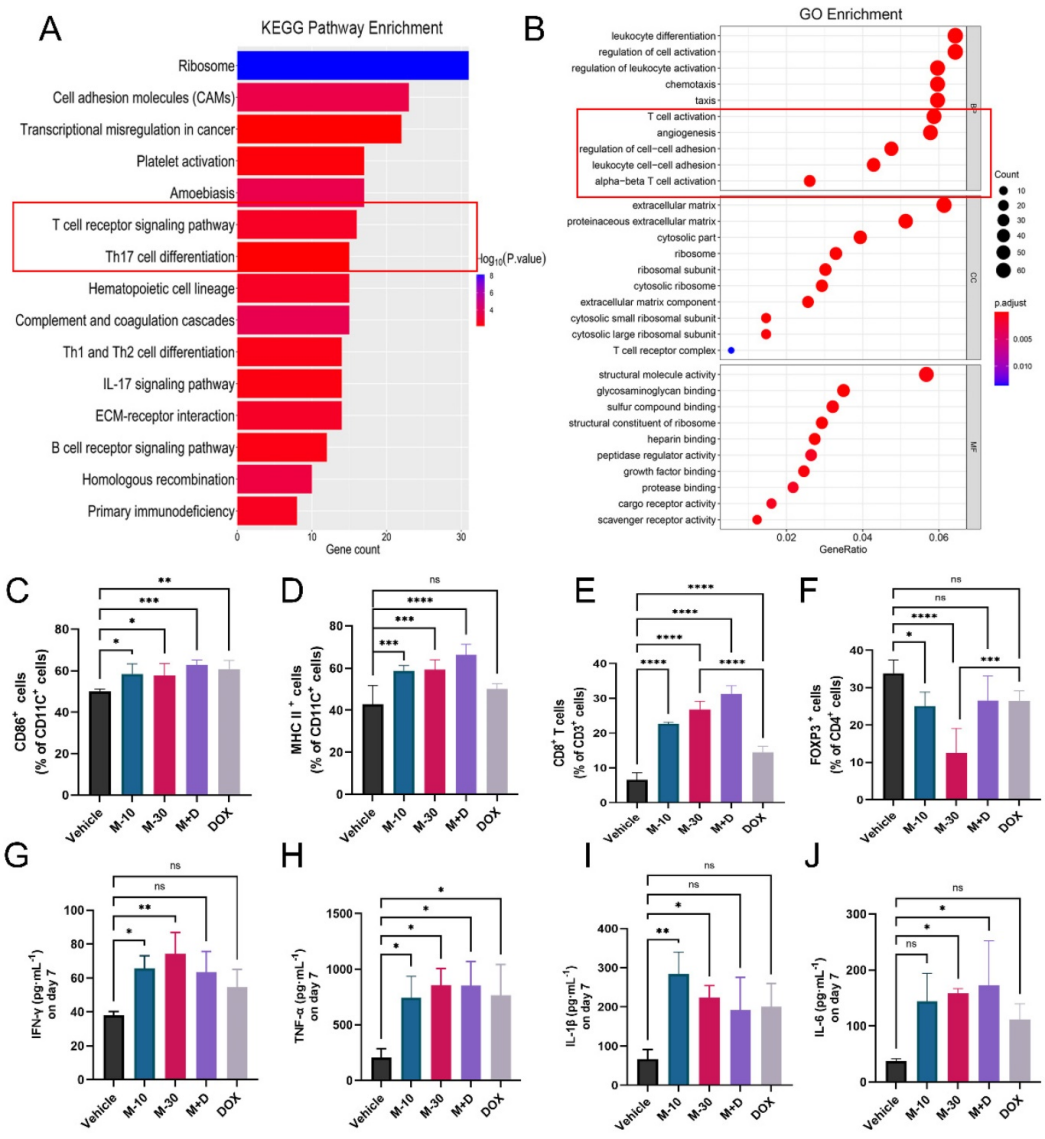
** MHO7 activated the antitumor immune response in mice. (A)** KEGG pathway enrichment of DEGs was analyzed by RNA-seq in 4T1 tumor tissue. **(B)** GO enrichment of DEGs was analyzed by RNA-seq in 4T1 tumor tissue. **(C)** Percentages of CD11C^+^/CD86^+^ DCs were measured by flow cytometry in spleens. **(D)** Percentages of CD11C^+^/ MHC II^+^ DCs were measured by flow cytometry in spleens. **(E)** Percentages of CD3^+^/CD8^+^ T cells were measured by flow cytometry in spleens. **(F)** Percentages of CD4^+^/FOXP3^+^ T cells were measured by flow cytometry in spleens. (n=6) The level of **(G)** IFN-γ, **(H)** TNF-α, **(I)** IL-1β, and** (J)** IL-6 were measured by ELISA in the serum on day 7 after treatment. Data was presented as mean ± SEM. *P <0.05; **P < 0.01; ***P <0.001; and ****P < 0.0001; ns=no significance.

Furthermore, total RNA sequencing was performed to measure the expression of genes in primary tumors in the M-10 group and vehicle group to clarify the effect of MHO7 on tumor-bearing mice with an intact immune system. There were 457 DEGs (|log2 (fold change)| >1, *P* < 0.05), with 369 upregulated genes and 88 downregulated genes. Notably, these DEGs mainly participated in multiple immune response pathways, especially T-cell signaling, as shown by KEGG pathway enrichment analysis, and regulated immune response functions, such as leukocyte differentiation and T-cell activation, as shown by GO enrichment analysis (Fig. 6A-6B). To explore changes in the immune system, splenocytes from tumor-bearing mice were collected and stained with DC and T-cell markers for analysis by flow cytometry. As a key indicator of immune activation, DCs first respond to DAMPs in the TME, linking innate and adaptive immune responses 38. Biomarkers of mature DCs (CD11C^+^/CD86^+^) in the treatment groups were significantly higher than those in the vehicle group (Fig. 6C, Fig. S5A). Major histocompatibility complex II (MHC-II) is regarded as one of the most important sites of antigen binding in DCs 39. The levels of CD11C^+^/MHC-II^+^ DCs increased to 62%, 65.3% and 70.4% in the M-10, M-30 and M+D groups, respectively. DOX alone activated 52.4% of DCs with no significant difference compared to 45.9% in the vehicle group (Fig. 6D, Fig. S5B). Moreover, in comparison to that in the vehicle group, a marked increase in the percentage of total T cells (CD45^+^/CD3^+^ T cells) was observed in the MHO7 treatment groups, and the ratio of helper T cells (CD3^+^/CD4^+^ T cells) was also considerably increased (Fig. S5E-S5F). Likewise, cytotoxic T cells (CD3^+^/CD8^+^ T cells) were increased in the M-10 (22.8%), M-30 (26.9%), and M+D (34%) groups but were reduced in the DOX group (13%) (Fig. 6E, Fig. S5C). In contrast, CD4^+^/FOXP3^+^ T cells, which are regulatory T cells (Tregs), were significantly decreased in mice in the M-10 (23.4%) and M-30 (9.49%) groups but showed no significant changes in the M+D (24.5%) and DOX (28.4%) groups compared to the vehicle group (Fig. 6F, Fig. S5D). On the other hand, serum specimens were collected from mice on Days 1, 7, and 14 after treatment, and antitumor cytokine levels (IFN-γ, TNF-α, IL-1β, and IL-6) were analyzed by ELISA. As shown in Fig. S6, the cytokines were increased by seven days and then decreased with time. We further compared changes in cytokines in the different groups on Day 7. MHO7-treated groups (M-10, M-30) had a higher level of IFN-γ than the vehicle group, but there was no significant difference in the M+D and DOX groups (Fig. 6G). Moreover, the level of TNF-α was enhanced in all treated groups on Day 7 (Fig. 6H). Next, the M-30 and M+D groups had higher secretion of IL-1β and IL-6 than the other groups (Fig. 6I and 6J). These results showed that MHO7 could effectively activate the antitumor immune response* in vivo.*

### MHO7 induced ICD* in vivo*

**Figure 7:**
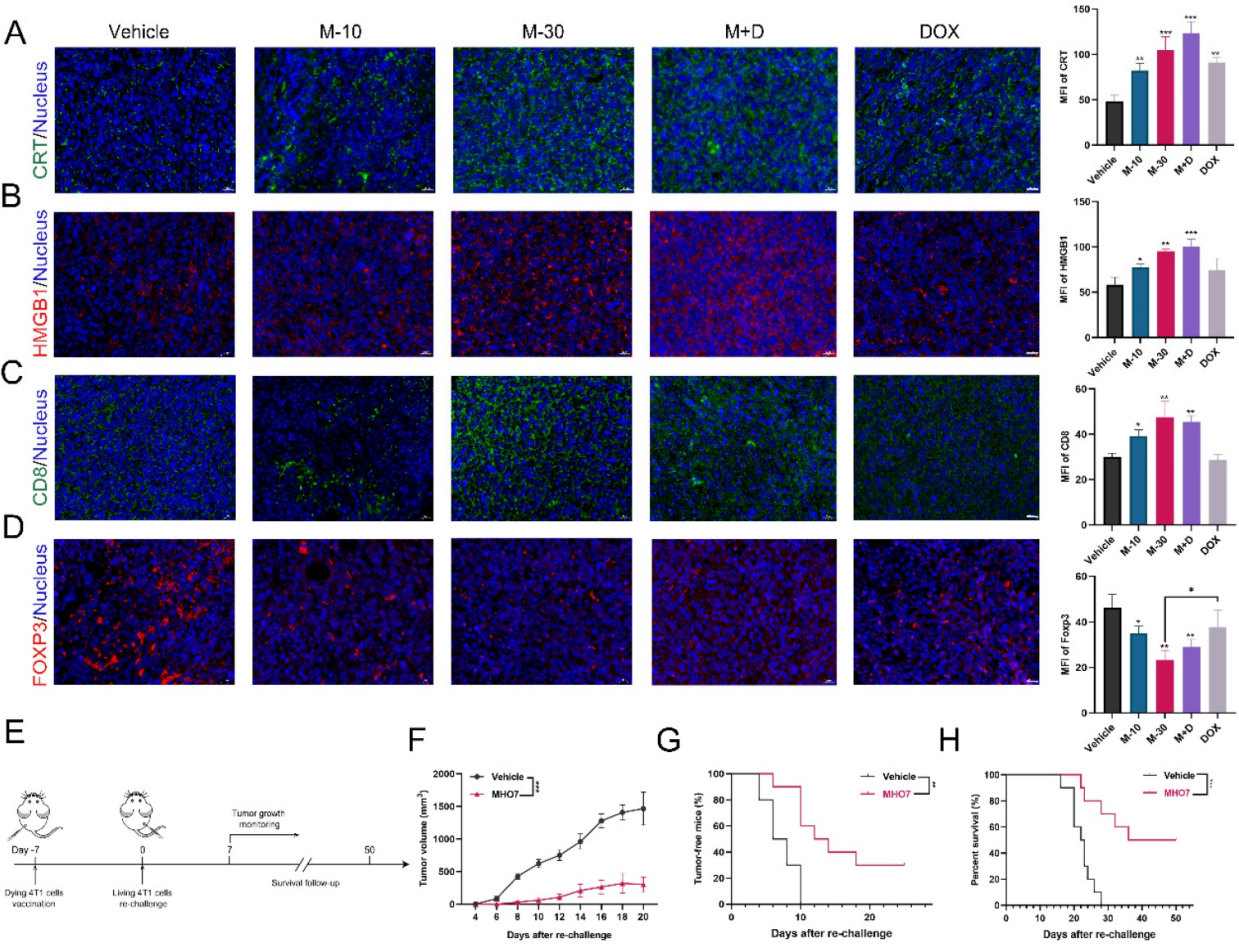
** MHO7 induced ICD *in vivo*. (A)** Tumor sections were stained with CRT (green) and DAPI (blue). **(B)** Tumor sections were stained with HMGB1 (red) and DAPI (blue). **(C)** Tumor sections were stained with CD8^+^ (green) and DAPI (blue). **(D)** Tumor sections were stained with FOXP3^+^ (red) and DAPI (blue). Scale bar, 20 µm. **(E)** Diagram of procedure for vaccination assay in mice. **(F)** Tumor growth was measured in mice. Data was presented as mean ± SEM. **(G)** Tumor-free progression was measured in mice. Log rank test. **(H)** Overall survival was measured of mice. Log rank test. *P <0.05; **P < 0.01; ***P <0.001; ns=no significant.

DAMPs that are released from cancer cells can facilitate antitumor therapy due to their connection with the immune system 16. Thus, the levels of CRT and HMGB1 in tumor tissues were examined. As Fig. 7A shows, MHO7 treatment led to a dramatic increase in CRT, and the combination of MHO7 and DOX induced a higher level of CRT than DOX alone. Moreover, the M-30 and M+D groups showed significantly elevated the levels of HMGB1, but the M-10 and DOX groups had no significant changes (Fig. 7B). Additionally, tumor-infiltrating CD8^+^ T cells were also increased in the MHO7-treated groups, while DOX did not effectively increase CD8^+^ T cells in tumors (Fig. 7C). In contrast, FOXP3 was significantly decreased by treatment with MHO7, but no significant changes were observed in response to DOX treatment (Fig. 7D). These results indicated that compared to DOX, MHO7 induced a stronger ICD-like antitumor immune response.

According to the consensus guidelines for ICD detection, vaccination is regarded as the gold standard to detect the ICD effect* in vivo* and assess the ability of treated cells to act as a tumor vaccine 40, 41. Thus, the rechallenged tumor model was established. 4T1 cells treated with vehicle or MHO7 were injected into the left flanks of mice, and untreated 4T1 cells were injected into the right flanks (Fig. 7E). It was observed that mice that were vaccinated with MHO7-treated cells had smaller tumor sizes than those in the vehicle group (Fig. 7F). Tumor incidence and the survival of mice were closely monitored in the following weeks. A total of 7/10 mice developed tumors in the MHO7-vaccinated group compared to 10/10 in the vehicle group after 18 days (Fig. 7G). Accordingly, MHO7-treated cells also significantly prolonged survival in rechallenged tumor models, suggesting that these cells could act as tumor vaccines *in vivo* (Fig. 7H). These results indicated that MHO7 could induce ICD *in vivo* and that MHO7-treated cells functioned effectively as a tumor vaccine.

## Discussion and Conclusion

MHO7 is a member of the Ophs family and is isolated from a marine fungus fermentation product. Studies have shown that Ophs exert antitumor effects by interacting with various organelles, including the ER. For example, it was reported that Oph A could induce paraptosis-like cell death characterized by swelling and fusion of mitochondria and/or the ER by inhibiting big conductance Ca^2+^-activated K^+^ channel (BKCa) activity in glioblastoma cells 42. Moreover, Oph A covalently modifies free sulfhydryl groups on proteins that may cause CHOP-mediated ER stress in human glioblastoma cells 43. These studies suggested that the ER might play a key role in the antitumor effect of MHO7. In this study, transcriptome analyses revealed that MHO7 caused a predominant change in the transcription of genes related to protein processes in the ER, suggesting that there was an interaction between MHO7 and the ER. In response to ER stress, BiP dissociates from PERK, and PERK undergoes transphosphorylation, subsequently phosphorylating eIF2α and activating the downstream signaling pathway 44, 45. Here, MHO7 promoted the expression of p-PERK and p-eIF2α (Fig. 2D and Fig. S2D), which indicated that BiP/GRP78 dissociated from PERK in response to MHO7 treatment. Then, MHO7 could induce apoptosis through the ER stress-mediated PERK/eIF2α/ATF4/CHOP pathway and CHOP played a major role in MHO7-induced apoptosis.

Presently, known ICD inducers are limited to certain chemotherapy agents (such as DOX, mitoxantrone, cyclophosphamide or oxaliplatin) 46 or physical treatments, such as radiotherapy and Hyp-PDT therapy 47. However, only a few small molecules have shown ICD effects 48. The activation of ICD relies on the combined action of ER stress and ROS generation and requires the production of sufficient DAMPs in the TME 49. In our study, we found that MHO7 could induce both ER stress and ROS production, and this function facilitated the release of ICD-related DAMPs. Here, ROS generation induced by MHO7 resulted in a low GSH/GSSG ratio, suggesting that MHO7 activated oxidative stress in TNBC cells. The high level of ROS also contributed to the apoptotic effect of MHO7. Additionally, severe ER stress accompanied by oxidative stress in response to MHO7 treatment led to the exposure of multiple DAMPs, including CRT, HMGB1, HSP70 and ATP, on the surface of TNBC cells.

In tumor-bearing mice, MHO7 inhibited tumor growth and metastasis, and the RNA-seq results revealed that the effect of MHO7 on tumor tissue was associated with T-cell activation. As an identified ICD inducer, it was reported DOX could be effectively phagocytosed by DCs to elicit cytotoxic T cells, inducing robust ICD *in vitro* or *in vivo* 37, 50. In response to the safe dose of DOX, we used DOX as a positive control to ensure ICD induced by MHO7* in vivo*. Accordingly, it was discovered that MHO7 had a similar effect as DOX on the activation of CD86^+^ DCs and CD4^+^ and CD8^+^ T cells but a more robust promoting effect on MHC-II DCs and an inhibitory effect on Tregs (FOXP3^+^ T cells)* in vivo*. Moreover, MHO7 triggered increased levels of IFN-γ, IL-1β and IL-6 than DOX and exerted a similar effect on TNF-α release, which indicated the more potent ICD-induced effect of MHO7 than DOX. In addition, MHO7 also showed a stronger tumor-specific ICD effect than DOX, as indicated by the higher expression of HMGB1 and CD8 and lower expression of FOXP3 in tumor tissue in response to MHO7 treatment. MHO7-treated cells functioned effectively as a tumor vaccine. These data suggested that MHO7 exerts a stronger ICD effect than DOX, activating a robust antitumor response to suppress TNBC growth and metastasis. It is worth pointing out that the anticancer activities of MHO7 are not limited to TNBC, but MHO7 would be more effective in high immune infiltration cancers such as TNBC.

To conclude, this study demonstrated that MHO7, a marine-derived sesterterpene compound, induced ICD through the ER stress-CHOP pathway. ER stress and ROS generation induced by MHO7 led to an ICD-mediated antitumor immune response* in vivo*. This therapeutic mechanism of MHO7 could not only suppress tumor growth but also improve the immunogenic ability of cancer cells to elicit a sustained immune response, providing a new strategy for TNBC treatment.

## Supplementary Material

Supplementary figures and table.

## Abbreviations

ATP: adenosine-5′-triphosphate
BKCa: big conductance Ca2+-activated K+ channel
MHO7: 6-epi-ophiobolin G
CHOP: C/EBP-homologous protein
CRT: calreticulin
CTL: Cytotoxic T lymphocyte
DAMPs: damage-associated molecular patterns
DC: Dendritic cells
DEGs: differentially expressed genes
DOX: doxorubicin
ER: endoplasmic reticulum
GSH: Glutathione
HMGB1: high mobility group protein B1
HSPs: heat shock proteins
ICBs: immune checkpoint blockers
ICD: immunogenic cell death
IFNγ: Interferonγ
IHC: Immunohistochemistry
IL: Interleukin
MHC-II: Major histocompatibility complexes class II
MMPs: mitochondrial membrane potentials
NAC: N-acetylcysteine
NSCLC: non-small-cell lung cancer
OphA: Ophiobolin A
Ophs: ophiobolins
OS: Overall survival
PDT: photodynamic therapy
PTT: photothermal therapy
ROS: Reactive oxygen species
TME: tumor microenvironment
TNBC: Triple-negative breast cancer
TNF-α: tumor necrosis factor-α
