# Supplementary material for: A marine-derived small molecule induces immunogenic cell death against triple-negative breast cancer through ER stress-CHOP pathway
Source: Int J Biol Sci. 2022 Apr 11;18(7):2898–913. doi: 10.7150/ijbs.70975 (PMC9066120; doi:10.7150/ijbs.70975)
Supplement: Supplementary file 1 — Supplementary figures and table. [file ijbsv18p2898s1.pdf]

Methods:

1. SUPPLEMENTARY TABLES

Table S1. Primer information of qRT-PCR

Table1 Primer information of qRT-PCR

Gene names	Primer names	Sequence	Length
GAPDH	GAPDH-F	TGGTATCGTGGAAGGACTC	19
	GAPDH-R	GGATGATGTTCTGGAGAGC	19
PERK	PERK-F	TCTTGGTCCCACTGGAAGAG	20
	PERK-R	AAAGCAGTGGGATTTGGATG	20
ATF3	ATF3-F	GTGCCGAAACAAGAAGAAGG	20
	ATF-R	TCTGAGCCTTCAGTTCAGCA	20
ATF4	ATF4-F	GTGTTCTCTGTGGGTCTGCC	20
	ATF4-R	GAGCCTCGTTCTTCTTTTCC	20
DDIT3	DDIT3-F	GACCCTGCTTCTCTGGCTTG	20
	DDIT3-R	CCGTTTCCTGGTTCTCCCTT	20
BAX	BAX-F	TCTGACGGCAACTTCAACTG	20
	BAX-R	GGGACATCAGTCGCTTCAGT	20
BCL2	BCL-2-F	GTGTGTGGAGAGCGTCAACC	20
	BCL2-R	AGCCAGGAGAAATCAAACAG	20

Results:

2 SUPPLEMENTARY FIGURES

Figure S1

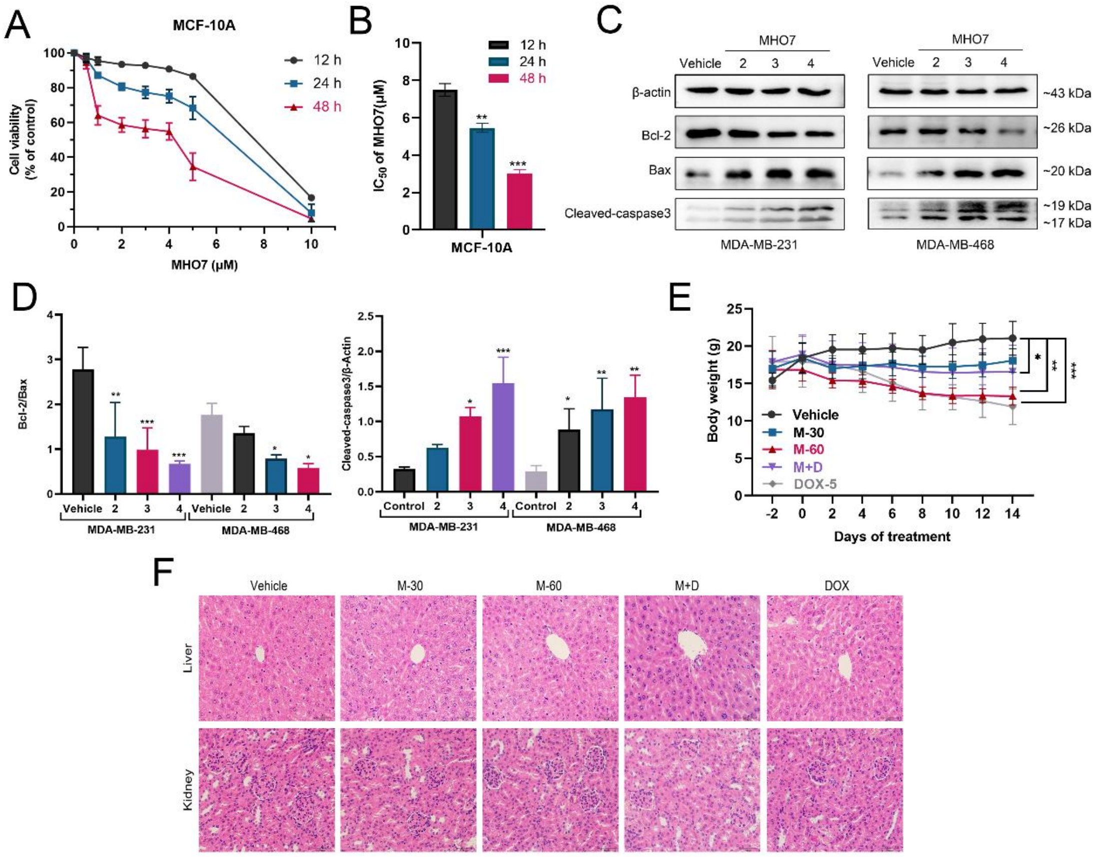

Figure S1. Antitumor effect of MHO7 *in vitro* and *in vivo.* (A) The effect of MHO7 on MCF-10A cells at 12, 24, and 48 h. **(B)** The IC_{50} value of MHO7 on MCF-10A at 12, 24, and 48 h. **(C-D)** The expression of Bxl2-2, Bax and cleaved caspase 3 were measured by western blot after the treatment of MHO7. **(E)** Body weight-time curves were measured in different groups. **(F)** Liver and kidney tissue in mice were analyzed H&E staining.

Figure S2

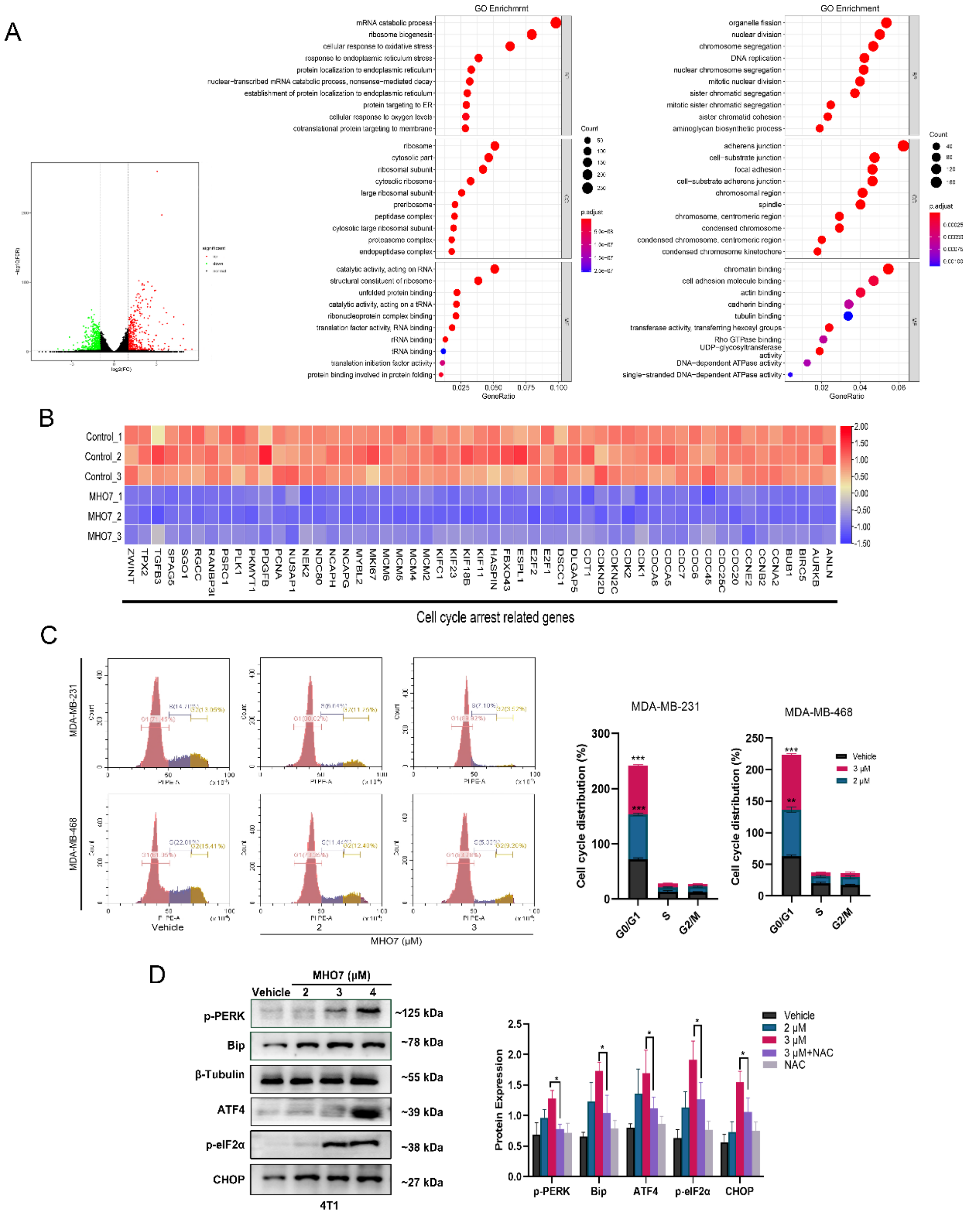

Figure S2. RNA-seq analysis revealed the induction of ER stress and cell cycle arrest in MHO7 treated MDA-MB-231 cells. (A) Volcano Plot and GO enrichment of DEGs were analyzed by RNA-seq. **(B)** Heat map of differentially expressed cell cycle-related genes was analyzed by RNA-seq. **(C)** Cell cycle was detected by flow cytometry after MHO7 treatment. **(D)** The expression of BiP/p-PERK/p-eIF2α/ATF4/CHOP were measured by western blot under MHO7 treatment in 4T1 cells.

Figure S3

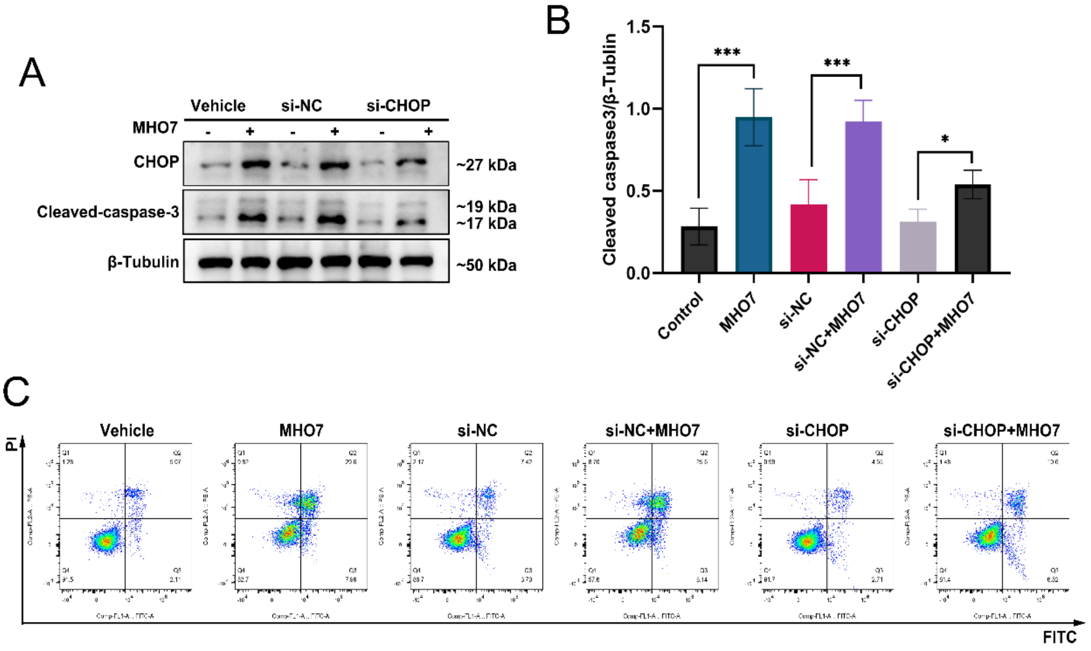

Figure S3. CHOP contributed to the apoptosis induced by MHO7. (A-B) CHOP and caspase3 expression measured by western blot in MDA-MB-231 cells after MHO7 (3 µM) or si-CHOP treatment. **(C)** The percentages of apoptosis cells were measured by flow cytometry under MHO7 (3 µM) or si-CHOP treatment. **P <0.05; **P < 0.01; ***P <0.001; and**** P < 0.0001.*

Figure S4

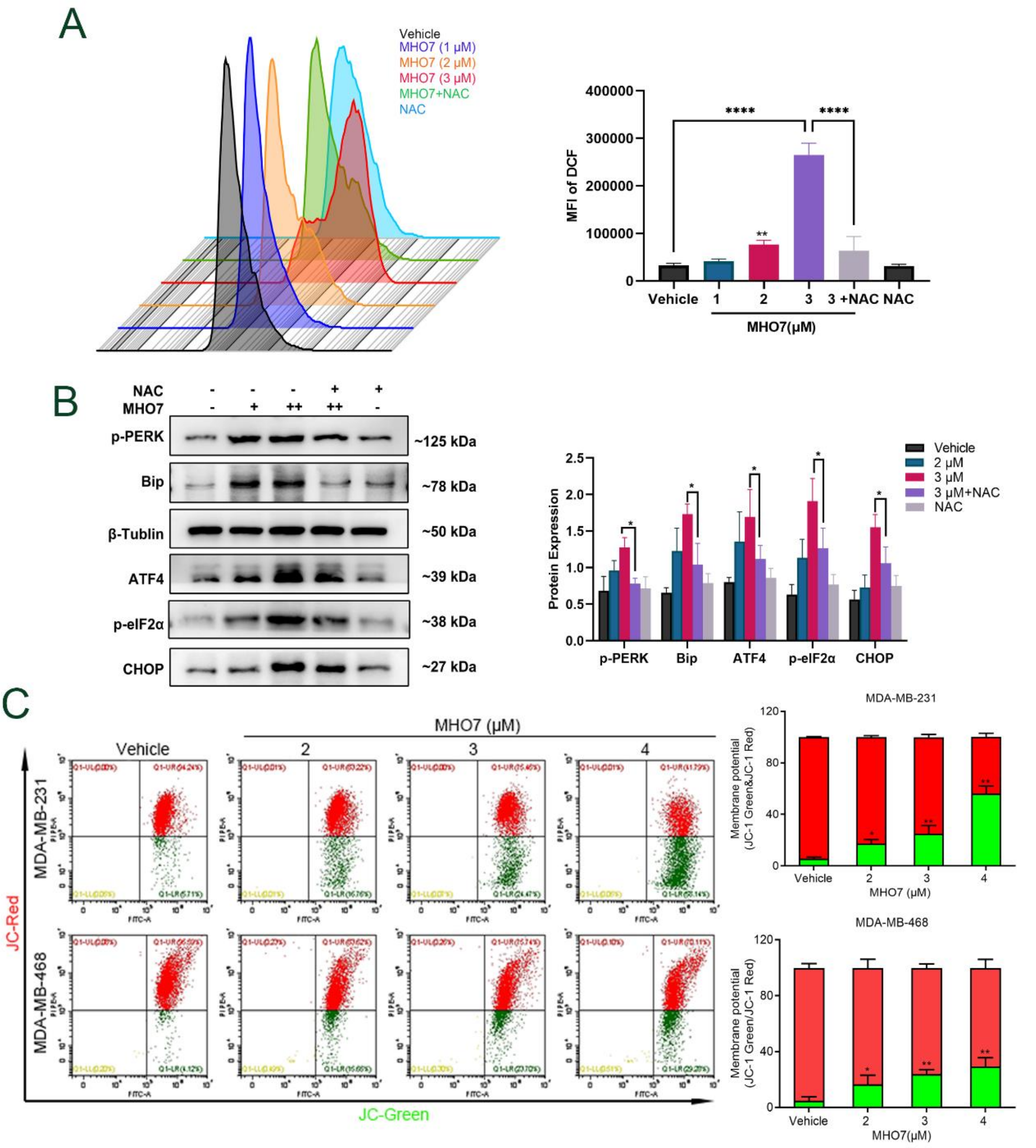

Figure S4. ROS generation and mitochondrial dysfunction induced by MHO7. (A) The ROS level was detected by flow cytometry when treated with MHO7 for 24 h or pretreated with NAC (4 mM) for 1 h in 4T1 cells. **(B)** ER stress-related proteins were measured by western blot under the treatment of MHO7 (+: 2 μM; ++:3 μM) or pretreated with NAC (4 mM) in 4T1 cells. **(C)** Mitochondrial membrane potentials (MMPs) were detected by flow cytometry after the treatment of MHO7. **P <0.05; **P < 0.01;*

Figure S5

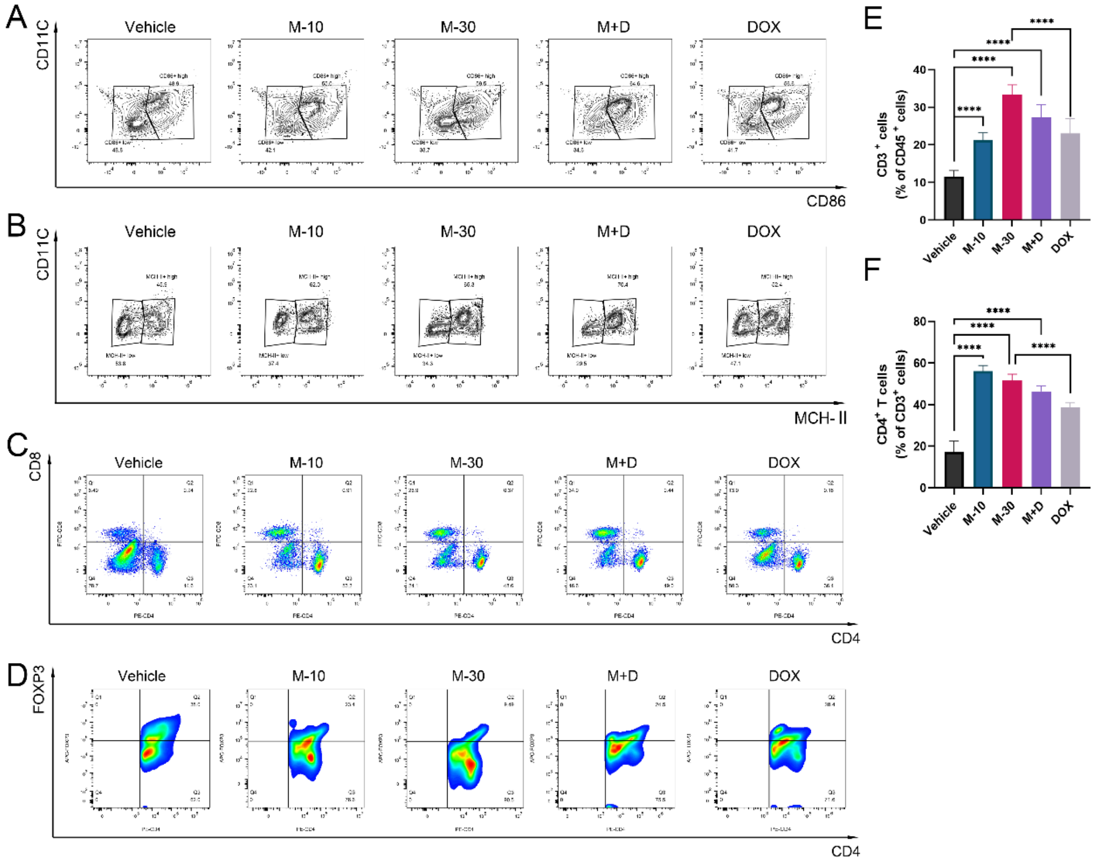

Figure S5. Maturation status of dendritic cells, activation of T cells and level of Tregs in MHO7 treated mice were analyzed by flow cytometry, respectively. (A) CD11C+/CD86+ DC cells in spleens were analyzed by flow cytometry. **(B)** CD11C+/ MHC II DC cells in spleens were analyzed by flow cytometry. **(C)** $CD3^+/CD8^+$ T cells in spleens were analyzed by flow cytometry. **(D)** $CD4^+/FOXP3^+$ T cells in spleens were analyzed by flow cytometry. **(E)** $CD3^+/CD8^+$ T cells in spleens were analyzed by flow cytometry. **(F)** $CD3^+$ /$CD4^+$ T cells were analyzed by flow cytometry. in spleens. **P <0.05; **P < 0.01; ***P <0.001; and**** P < 0.0001.*

Figure S6

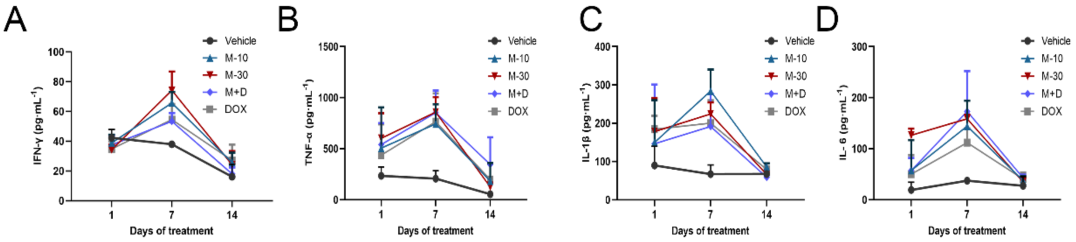

Figure S6. Antitumor cytokine levels, including (A) IFN-γ, (B) TNF-α, (C) IL-1β and (D) IL-6 in the serum were detected by ELISA from each group on day 1,7,14 after treatment. (n=3).
